# A novel method for intratunnel closure of mucosal injuries during peroral endoscopic myotomy using standard endoclips

**DOI:** 10.1055/a-2107-2999

**Published:** 2023-07-13

**Authors:** Ahmad Madkour, Amr Elfouly, Osama Elnahas, Sara Moreed, Hassan Atalla

**Affiliations:** 1 Endemic Medicine Department, Faculty of Medicine, Helwan University, Cairo, Egypt; 2 Faculty of Medicine, Helwan University, Cairo, Egypt; 3 Internal Medicine, Dar Shefa Hospital, Cairo, Egypt; 4 Hepatology and Gastroenterology Unit, Department of Internal Medicine, Faculty of Medicine, Mansoura University, Mansoura, Egypt

Mucosal perforations, reported in 4.2 %–17.3 % of cases 1 , are one of the major inadvertent complications of peroral endoscopic myotomy. Mostly, mucosal perforations occur at the gastroesophageal junction (GEJ), where the muscularis propria and mucosal layers are in close proximity, representing major challenges for endoscopic management 2 . This is largely related to the constrained position of the endoscope and the challenge of controlling the direction of the endoclips, particularly when using the retroflexed view below the cardia. Over-the-scope clips 3 , endosutures 4 , fibrin sealant 1 , and even diluted cyanoacrylate 5 have been reported as being used in such situations, yet they are not usually available and their costs are high.

Here, we demonstrate two successful cases using a unique and simple method for the closure of mucosal perforations using standard endoclips ( Video 1 ). We used the previously created submucosal tunnel to provide a convenient space for easier application of endoclips, with better malleability and fewer numbers needed. Moreover, this was applicable both before and after endoscopic myotomy.

**Video 1** A simple and unique method for intratunnel closure of mucosal injuries during peroral endoscopic myotomy using conventional endoclips.


Both patients had a prior history of either Heller’s myotomy or endoscopic dilation, leaving extensive fibrosis at the GEJ, which resulted in difficult dissection and inadvertent mucosal perforation. In the first patient, the endoclip was applied after selective myotomy had been performed, because of the fear of further limiting the dissection space ( Fig. 1 ). In the other patient, the endoclip was applied before myotomy (our recommendation), with the aim of avoiding any further unintended extension of the mucosal perforation during myotomy ( Fig. 2 ). Both patients had uneventful follow-ups, with significant clinical improvement. Follow-up endoscopies revealed complete healing of the mucosal perforation in patient #1 ( Fig. 3 ); however, in patient #2, alongside complete healing, the endoclip was found hanging at the GEJ ( Fig. 4 ), which might be attributed to progressive narrowing of the healing intratunnel space. The endoclip was easily removed, without any adverse events.

**Fig. 1:**
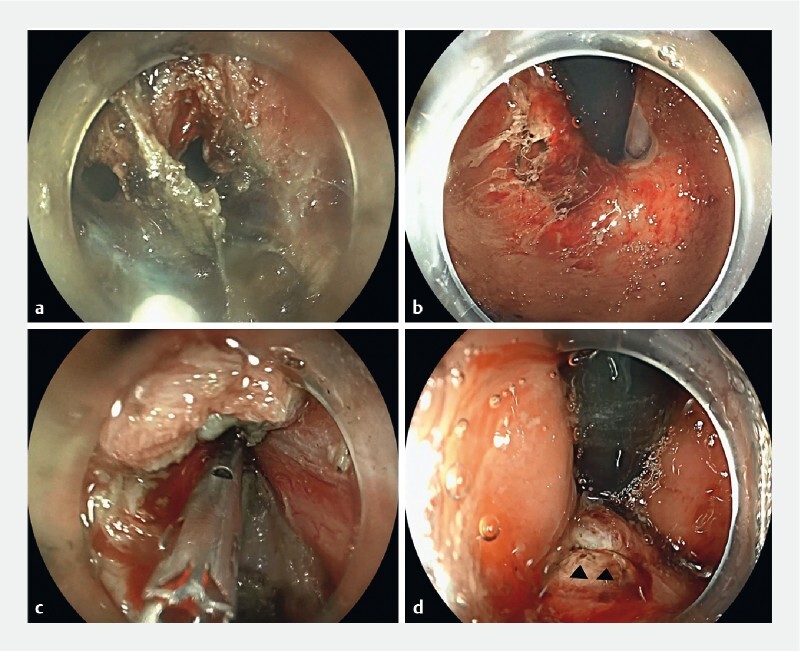
Endoscopic images in patient #1 showing: **a** the mucosal perforation at the gastroesophageal junction from inside the tunnel; **b** the same mucosal perforation from the gastric side; **c** successful intratunnel complete closure of the perforation using a single standard endoclip; **d** linear appearance of the perforation (black arrowheads) from the gastric side.

**Fig. 2:**
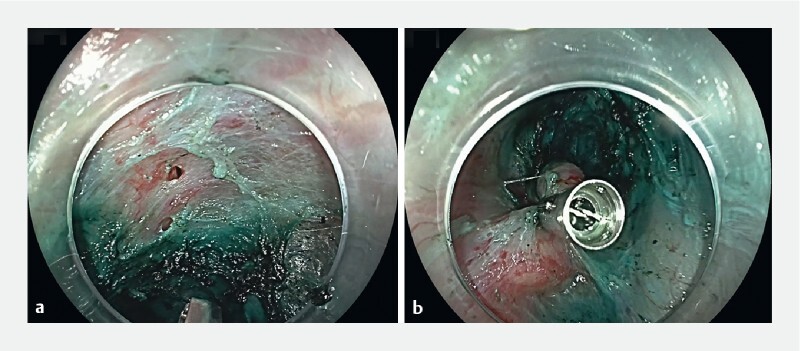
Endoscopic images in patient #2 showing: **a** the mucosal perforation at the gastroesophageal junction from inside the tunnel; **b** successful intratunnel complete closure of the perforation using a single standard endoclip.

**Fig. 3:**
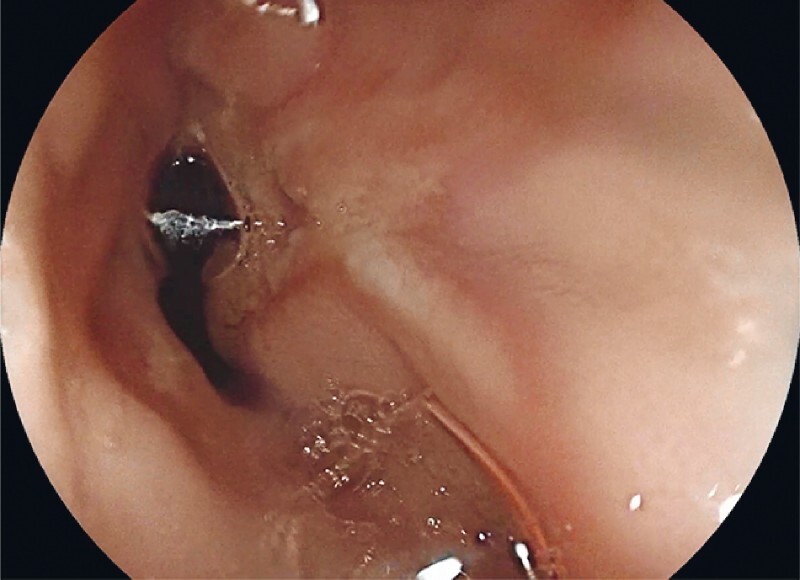
Image from follow-up endoscopy a few months after the procedure showing complete healing of the mucosal perforation in patient #1.

**Fig. 4:**
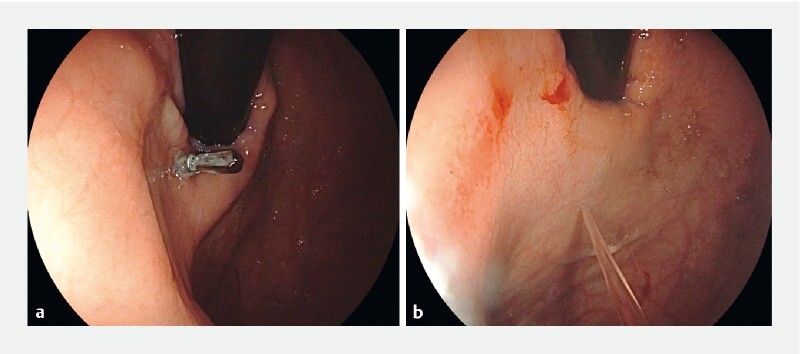
Image from follow-up endoscopy in patient #2 showing: **a** complete healing of the perforation, but with the endoclip hanging at the gastroesophageal junction, surprisingly, with the jaws directed into luminal side; **b** appearance of the healed perforation after the endoclip was easily removed.

To the best of our knowledge, this is the first report of this intratunnel technique, which seems to be feasible, easier, and less costly than the other previously mentioned modalities and with comparable safety. Evaluation of the long-term outcomes needs further large-scale studies.

Endoscopy_UCTN_Code_TTT_1AO_2AG

## Notes

Endoscopy E-Videos

https://eref.thieme.de/e-videos
*E-Videos* is an open access online section of the journal *Endoscopy* , reporting on interesting cases and new techniques in gastroenterological endoscopy. All papers include a high-quality video and are published with a Creative Commons CC-BY license. Endoscopy E-Videos qualify for HINARI discounts and waivers and eligibility is automatically checked during the submission process. We grant 100% waivers to articles whose corresponding authors are based in Group A countries and 50% waivers to those who are based in Group B countries as classified by Research4Life (see: https://www.research4life.org/access/eligibility/ ). This section has its own submission website at https://mc.manuscriptcentral.com/e-videos
